# Supplementary material for: Merging toroidal dipole bound states in the continuum without up-down symmetry in Lieb lattice metasurfaces
Source: Nanophotonics. 2024 Mar 8;13(9):1561–8. doi: 10.1515/nanoph-2023-0686 (PMC11636481; doi:10.1515/nanoph-2023-0686)
Supplement: Supplementary file 1 — Supplementary Material Details [file j_nanoph-2023-0686_suppl_001.pdf]

# Supporting Information

# Merging toroidal dipole bound states in the continuum without up-down symmetry in Lieb lattice metasurfaces

*Guodong Zhu[1,2], Sen Yang[1,3], Justus C. Ndukaife[1,2,3*]*

[1]Department of Electrical and Computer Engineering, Vanderbilt University, Nashville, TN, USA 37235

[2]Vanderbilt Institute of Nanoscale Science and Engineering, Vanderbilt University, Nashville, TN, USA 37235

3Interdisciplinary Materials Science, Vanderbilt University, Nashville, TN, 37235, USA

‡These authors contribute equally to this work.

*Correspondence: justus.ndukaife@vanderbilt.edu

Merging toroidal dipole bound states in the continuum without up-down symmetry in Lieb lattice metasurfaces

## 1. Multipole characters of the toroidal dipole mode

We utilized multipolar decomposition based on Cartesian coordinates as an analytical technique to investigate the multipole component of the mode in TM2 band [1-5]. Initially, we computed the polarization based on the near-field distribution near the resonator. The near-field distributions were obtained using a commercially available electromagnetic solver called COMSOL.

The Polarization, $\mathbf{P}(\mathbf{r})$, is expressed as follows:

$$\mathbf{P}(\mathbf{r}) = \varepsilon_0(\varepsilon_r - \varepsilon_d)\mathbf{E}(\mathbf{r}) \tag{1}$$

,where $\varepsilon_0$ is the permittivity of free space, and $\varepsilon_r$ and $\varepsilon_d$ are the relative dielectric permittivity of the resonators and surrounding medium, respectively. $\mathbf{E}(\mathbf{r})$ is the total electric field inside the resonators. The first five orders of the multipolar components, including electric dipole ($p$),

magnetic dipole ($m$ ), electric quadrupole ($\hat{Q}^e$), magnetic quadrupole ($\hat{Q}^m$), and toroidal dipole ($t$ ) are expressed by:

$$p = \int_V \boldsymbol{P}(\boldsymbol{r})d\boldsymbol{r} \tag{2}$$

$$m = \frac{i\omega}{2}\int_V [\boldsymbol{r} \times \boldsymbol{P}(\boldsymbol{r})]d\boldsymbol{r} \tag{3}$$

$$\hat{Q}^e = 3\int_V [\boldsymbol{r}\boldsymbol{P}(\boldsymbol{r}) + \boldsymbol{P}(\boldsymbol{r})\boldsymbol{r} - \frac{2}{3}(\boldsymbol{r} \cdot \boldsymbol{P}(\boldsymbol{r}))\hat{U}]d\boldsymbol{r} \tag{4}$$

$$\hat{Q}^m = \frac{\omega}{3i}\int_V \{[\boldsymbol{r} \times \boldsymbol{P}(\boldsymbol{r})]\boldsymbol{r} + \boldsymbol{r}[\boldsymbol{r} \times \boldsymbol{P}(\boldsymbol{r})]\}d\boldsymbol{r} \tag{5}$$

$$t = \frac{i\omega}{10}\int_V \{[2(\boldsymbol{r})^2 \cdot \boldsymbol{P}(\boldsymbol{r})]\boldsymbol{r} + [\boldsymbol{r} \cdot \boldsymbol{P}(\boldsymbol{r})]\boldsymbol{r}\}d\boldsymbol{r} \tag{6}$$

where $\boldsymbol{r}$ is the radius vector of a volume element inside the resonator, $\omega$ is the circular frequency, $\hat{U}$ is the $3 \times 3$ unit sensor.

This multipolar analysis contains the electric field information contributed by four silicon resonators. The results from the multipole decomposition analysis are presented in Figures 2a and 2b in the main manuscript.

## 2. Robustness of the merging design

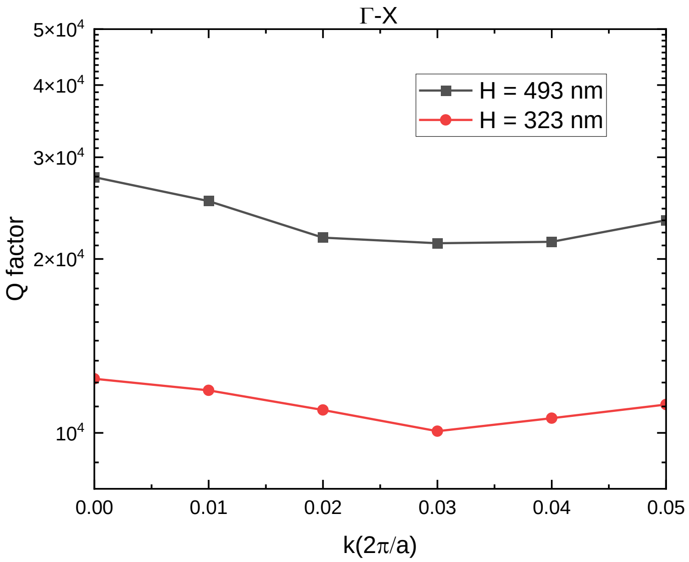


Figure S1. The simulated Q factor near the center of the Brillouin zone, obtained from a $15 \times 15$ perturbated supercell, is shown for both mering and isolated BIC, respectively. The perturbations are applied on positions as well as the radius. The black line shows the Q factor evolution of merging BIC in momentum space while the red line presents the Q factor evolution of single BIC. Notably, this visualization reveals that merging BICs maintain a relatively high Q factor even in the presence of disorders and imperfections.

## 3. The Q factor varies with the size of the BIC region

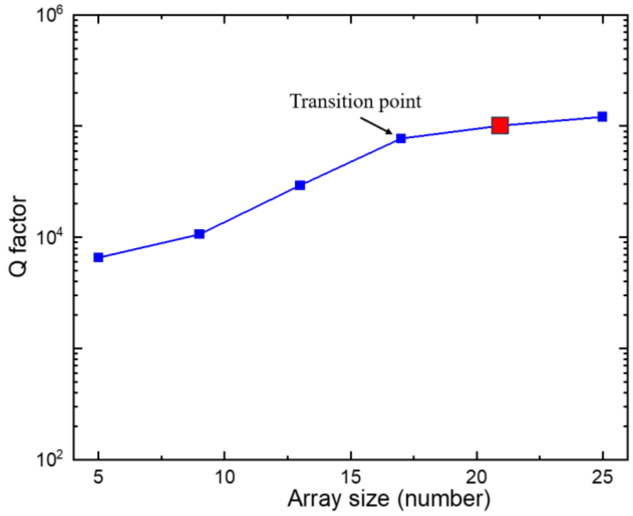


Figure S2. The blue line shows Q factor changes with array size of BIC region which is encapsulated by the band gap mirror. We selected $21 \times 21$ array size (mark as red) in our simulation as the size is close to the transition point and the Q factor is up to $10^5$.

## 4. The Q factor varies with the thickness of the mirror

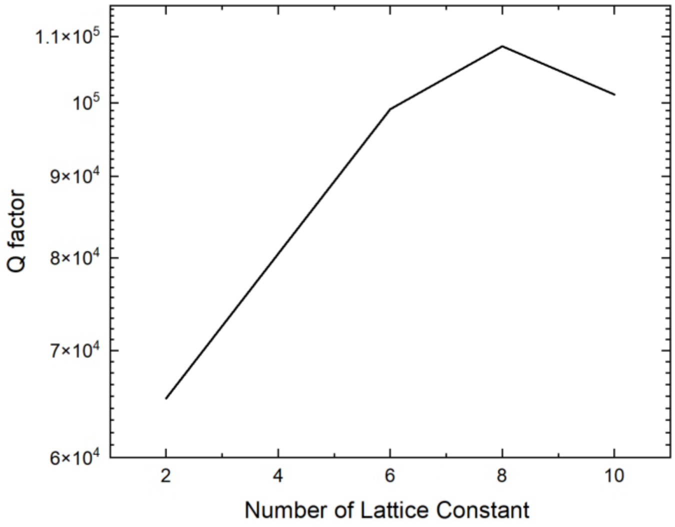


Figure S3. The figure shows that Q factor of the finite size metasurface changes with the "thickness" of the mirror structure. Thickness is measured by the lattice constant for the mirror.

## 5. Forming a complete accidental BIC in photonics crystal slabs without up-down symmetry

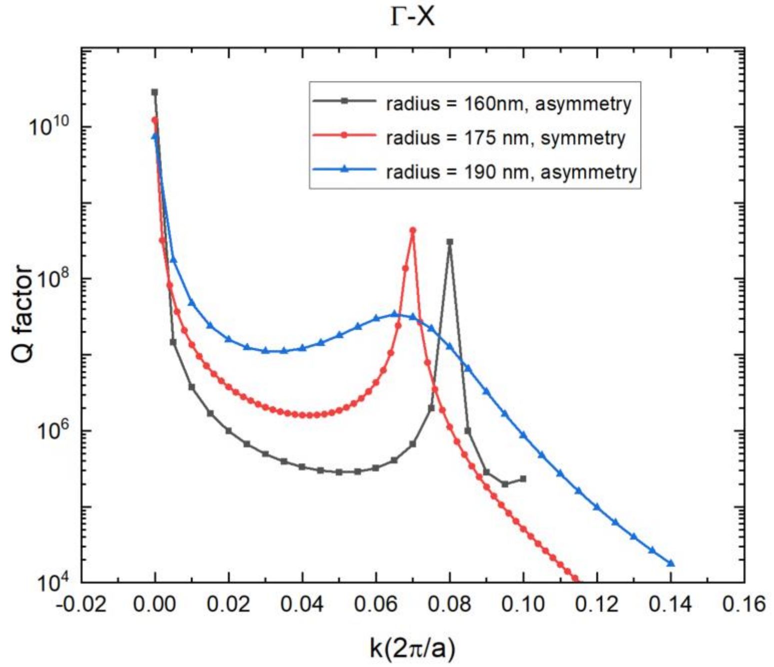


Figure S4. The simulation is based on the slab structure in [1]. The figure shows a complete accidental BIC can be achieved via tunning the radius of the hole. (a) shows that when the superstrate (refractive index =1.33) and substrate (refractive index = 1.46) are different, by increasing the height and radius of pillars, a complete accidental BIC can be achieved. (b) shows that when the superstrate and substrate (refractive index = 1.46) are the same, a complete accidental BIC can be achieved at lower height and radius of pillars.

## 6. Forming a complete accidental BIC via increasing height and radius of pillars

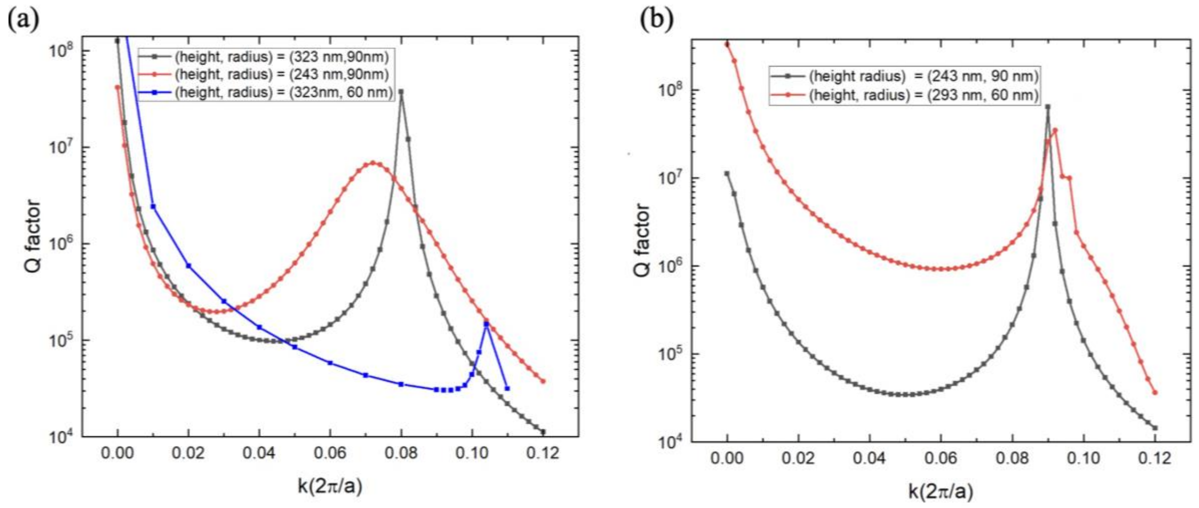

Figure S5. The Q factor of the accidental BIC changes with the height and radius of the metasurface. (a) shows that when the superstrate (refractive index =1.33) and substrate (refractive index = 1.46) are different, by increasing the height and radius of pillars, a complete accidental BIC can be achieved. (b) shows that when the superstrate and substrate (refractive index = 1.46) are the same, a complete accidental BIC can be achieved even at lower height and radius of pillars.

## 7. Band structure simulation for pillar structure in square lattice

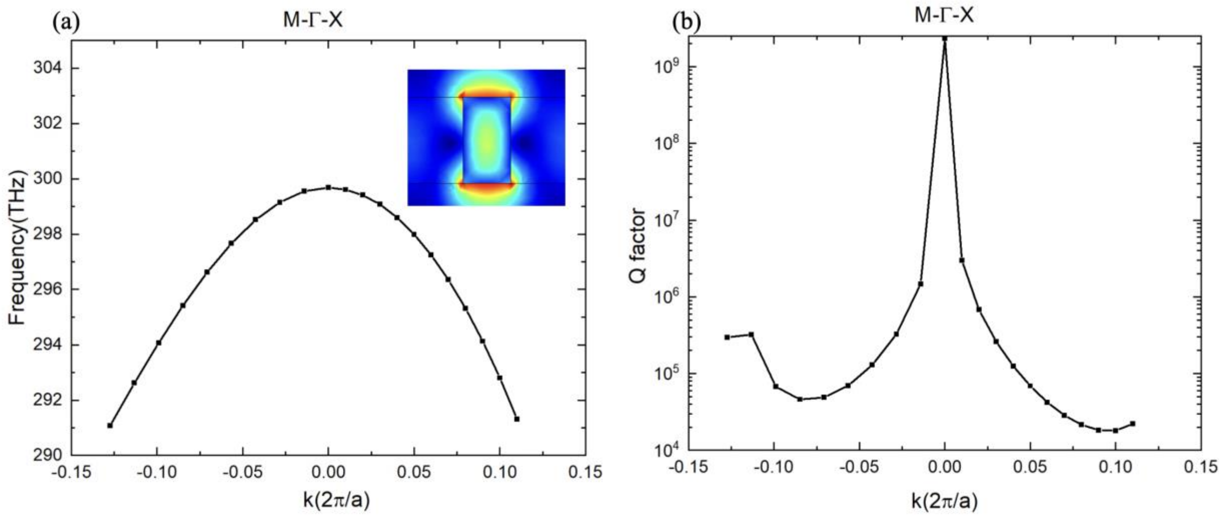


Figure S6. The band structure and Q factor variation in k space for the square lattice configuration. The insight in (a) shows the electric field distribution in the XZ plane. (b) Shows there is no accidental BIC in the Γ-X and Γ-M direction in k space.

# References

(1) Hinamoto, T.; Fujii, M. MENP: An Open-Source MATLAB Implementation of Multipole Expansion for Nanophotonics. *OSA Contin* **2021**, *4* (5), 1640. https://doi.org/10.1364/OSAC.425189.

(2) Ray, D.; Raziman, T. V.; Santschi, C.; Etezadi, D.; Altug, H.; Martin, O. J. F. Hybrid Metal-Dielectric Metasurfaces for Refractive Index Sensing. *Nano Lett* **2020**, *20* (12), 8752–8759. https://doi.org/10.1021/acs.nanolett.0c03613.

(3) Li, Q.; van de Groep, J.; White, A. K.; Song, J. H.; Longwell, S. A.; Fordyce, P. M.; Quake, S. R.; Kik, P. G.; Brongersma, M. L. Metasurface Optofluidics for Dynamic Control of Light Fields. *Nat Nanotechnol* **2022**, *17* (10), 1097–1103. https://doi.org/10.1038/s41565-022-01197-y.

(4) Moretti, G. Q.; Tittl, A.; Cortés, E.; Maier, S. A.; Bragas, A. V.; Grinblat, G. Introducing a Symmetry-Breaking Coupler into a Dielectric Metasurface Enables Robust High-Q Quasi-BICs. *Adv Photonics Res* **2022**, 2200111. https://doi.org/10.1002/adpr.202200111.

(5) Li Z, Wu T, Zhang X. Tailoring toroidal and magnetic dipole excitations with the same dielectric structure. Opt Lett. 2019 Jan 1;44(1):57-60. doi: 10.1364/OL.44.000057. PMID: 30645547.

(6) Jin, J., Yin, X., Ni, L. et al. Topologically enabled ultrahigh-Q guided resonances robust to out-of-plane scattering. Nature 574, 501–504 (2019). https://doi.org/10.1038/s41586-019-1664-7
